# Indicações terapêuticas em demandas judiciais por *Cannabis* medicinal no Brasil: um estudo transversal perante as evidências científicas

**DOI:** 10.1590/0102-311XPT220525

**Published:** 2026-07-06

**Authors:** Cláudia Du Bocage Santos Pinto, Tatiana De Jesus Nascimento Ferreira, Catia Veronica dos Santos Oliveira, Ângela Esher, Marina Fajardo Villela Martins Pompilho da Hora, Claudia Garcia Serpa Osorio-de-Castro

**Affiliations:** 1 Faculdade de Medicina, Universidade Federal de Mato Grosso do Sul, Campo Grande, Brasil.; 2 Escola Nacional de Saúde Pública Sergio Arouca, Fundação Oswaldo Cruz, Rio de Janeiro, Brasil.; 3 Instituto de Matemática e Estatística, Universidade do Estado do Rio de Janeiro, Rio de Janeiro, Brasil.

**Keywords:** Cannabis sativa, Canabidiol, Sistema Único de Saúde, Judicialização da Saúde, Saúde Pública Baseada na Evidência, Cannabis sativa, Cannabidiol, Unified Health System, Healths’ Judicialization, Public Health Evidence-based, Cannabis sativa, Canabidiol, Sistema Único de Salud, Judicialización de la Salud, Salud Pública Basada en la Evidencia

## Abstract

Nos últimos anos, a acelerada expansão do mercado de produtos de *Cannabis* medicinal (PCM) levou ao aumento do consumo e das demandas judiciais pleiteando PCM no Brasil. O objetivo do estudo foi analisar as indicações terapêuticas presentes nas demandas judiciais que pleiteiam PCM, ajuizadas contra a União, e frente às evidências científicas presentes na literatura. Foi realizado um estudo transversal, analisando-se processos referentes aos pleitos depositados entre 2020 e 2023, e executada uma síntese qualitativa rápida de evidências (SQRE) relacionada às indicações da *Cannabis* medicinal apontadas nos processos. Nas 2.024 demandas, foram identificados 1.962 diagnósticos primários, sendo epilepsias (36,24%), transtorno do espectro autista (TEA) (25,54%) e dor (8,92%) as condições mais frequentes. Observou-se alta frequência de demandantes no intervalo etário de 1 a 19 anos para epilepsia, TEA, paralisias e deficiência intelectual. Na SQRE, foram resgatadas 115 revisões com emprego de ferramentas de avaliação de qualidade, que traziam informações sobre a efetividade de PCM, apontando dor (40,86%), neoplasia (10,43%) e epilepsia (9,56%) como condições mais estudadas. Para apenas cinco diagnósticos - epilepsia, esclerose múltipla, ansiedade, neoplasia e dor - houve algum suporte na literatura, em graus diferentes, e com frequência baixa de revisões de alta qualidade. A grande pressão da judicialização e a quantidade de indicações sem evidências destacam um risco sanitário considerável que não tem tido abordagem tempestiva no país. É urgente a regulamentação dos PCM e sua prescrição apropriada, resguardando o fornecimento público apenas àquelas baseadas em evidências robustas.

## Abstract (en)

In recent years, the rapid expansion of the medical *Cannabis* products (MCP) market has led to increased consumption and a rise in lawsuits seeking access to MCP in Brazil. This study aimed to analyze therapeutic indications cited in lawsuits filed against the Brazil’s Federal Government requesting MCP and to assess these indications in light of the available scientific evidence. This cross-sectional study was conducted analyzing cases filed between 2020 and 2023. Additionally, a rapid qualitative evidence synthesis (RQES) was performed addressing the medical *Cannabis* indications reported in the lawsuits. Across 2,024 lawsuits, 1,962 primary diagnoses were identified. The most frequent conditions were epilepsy (36.24%), autism spectrum disorder (ASD) (25.54%), and pain (8.92%). A high proportion of claimants in the 1 to 19 age range was observed for epilepsy, ASD, paralysis, and intellectual disabilities. The RQES retrieved 115 reviews that applied quality assessment tools and reported on the effectiveness of MCP. The most frequently studied conditions were pain (40.86%), neoplasms (10.43%), and epilepsy (9.56%). Only five diagnoses - epilepsy, multiple sclerosis, anxiety, neoplasms, and pain - had some degree of support in the literature, varying in strength and with a low proportion of high-quality reviews. The substantial pressure from health-related litigation and the large number of indications lacking supporting evidence highlight a significant public health risk that has not been addressed in a timely manner in Brazil. Regulation of MCP and appropriate prescribing practices are urgently needed, restricting public provision to indications supported by robust evidence.

## Abstract (es)

En los últimos años, la rápida expansión del mercado de productos de *Cannabis* medicinal (PCM) ha llevado a un aumento del consumo y de las demandas judiciales que reclaman PCM en Brasil. El objetivo del estudio fue analizar las indicaciones terapéuticas presentes en las demandas judiciales que reclaman PCM, presentadas contra la Unión, y compararlas con las pruebas científicas presentes en la literatura. Se realizó un estudio transversal en el que se analizaron los procesos relacionados con las demandas presentadas entre 2020 y 2023, y se llevó a cabo una síntesis cualitativa rápida de la evidencia (SQRE) relacionada con las indicaciones del *Cannabis* medicinal señaladas en los procesos. En las 2.024 demandas se identificaron 1.962 diagnósticos primarios, de los cuales los más frecuentes fueron epilepsia (36,24%), trastorno del espectro autista (TEA) (25,54%) y dolor (8,92%). Se observó una alta frecuencia de demandantes en el rango de edad de 1 a 19 años, por epilepsia, TEA, paralisis e discapacidad intelectual. En la SQRE se recuperaron 115 revisiones con el uso de herramientas de evaluación de la calidad, que aportaban información sobre la eficacia de los PCM, señalando el dolor (40,86%), la neoplasia (10,43%) y la epilepsia (9,56%) como las afecciones más estudiadas. Solo cinco diagnósticos -epilepsia, esclerosis múltiple, ansiedad, neoplasia y dolor- contaban con algún respaldo en la literatura, en distintos grados y con una baja frecuencia de revisiones de alta calidad. La gran presión de la judicialización y la cantidad de indicaciones sin evidencia ponen de manifiesto un riesgo sanitario considerable que no se ha abordado de manera oportuna en el país. Es urgente regular los PCM y su prescripción adecuada, garantizando el suministro público solo de aquellos basados en evidencias sólidas.

## Introdução

Nos últimos anos, o debate internacional sobre a legalização da *Cannabis* veio acompanhado da ampliação das pesquisas sobre a planta. A partir da década de 1980, estudos elucidaram mecanismos de ação dos fitocanabinoides ^1^ ^,^ ^2^. Em especial, investigações sobre o sistema endocanabinóide e seus receptores amplamente distribuídos no organismo impulsionaram hipóteses terapêuticas para diversas indicações ^3^ ^,^ ^4^ ^,^ ^5^. Entretanto, a expectativa de benefício em diversas condições levou à divulgação precoce de várias indicações, contribuindo para o aumento do consumo e para uma expansão acelerada do mercado de produtos de *Cannabis* medicinal (PCM) ^6^ ^,^ ^7^ ^,^ ^8^.

No Brasil, a política de fornecimento gratuito de medicamentos e tecnologias pelo Sistema Único de Saúde (SUS) envolve produtos que tenham sido registrados junto à Agência Nacional de Vigilância Sanitária (Anvisa) e que tenham sido avaliados pela Comissão Nacional de Incorporação de Tecnologias no Sistema Único de Saúde (Conitec). Tais etapas visam assegurar, além da eficácia, efetividade e segurança, a avaliação econômica comparativa, ponderando benefícios e custos frente às tecnologias já existentes ^9^.

O atual cenário sobre a *Cannabis* medicinal no Brasil envolve um único medicamento registrado junto à Anvisa (tetraidrocanabinol (THC) 27mg/mL + canabidiol (CBD) 25mg/mL) indicado para espasticidade na esclerose múltipla ^10^, com pedido de incorporação junto à Conitec em 2020. Além desse, outra tecnologia, não registrada como medicamento no Brasil, também teve incorporação solicitada: o CBD 200mg/mL para crianças e adolescentes com epilepsia refratária. Ambas as propostas foram negadas por evidências insuficientes ou de baixa qualidade/confiabilidade; assim, até o momento, nenhum PCM foi incorporado ao SUS ^11^.

Diante do contexto de não incorporação e à pressão social fomentada por um mercado emergente e de alta lucratividade ^12^, o número de demandas judiciais pleiteando PCM vem aumentando, já que a judicialização é hoje vista como uma via de acesso regular às tecnologias de saúde no SUS.

O objetivo deste trabalho foi analisar as indicações terapêuticas presentes nas demandas judiciais que pleiteiam *Cannabis* medicinal, ajuizadas contra o ente federal no Brasil e frente às evidências científicas presentes na literatura.

## Metodologia

Foi realizado um estudo transversal envolvendo demandas judiciais para o fornecimento de produtos contendo *Cannabis* para fins medicinais, impetradas contra a União, no Brasil. Foram consultados os processos depositados no Departamento de Gestão das Demandas em Judicialização na Saúde (DJUD) do Ministério da Saúde, de 2020 a 2023. 

### Identificação e coleta de dados dos processos

Os processos de interesse foram identificados pelo DJUD a partir das palavras-chave “cannabis”, “canabidiol”, “canabinoides”, “CBD”. Uma lista de processos pleiteando produtos contendo cannabis para fins medicinais foi gerada, excluindo aqueles relacionados à autorização para plantio, importação de sementes ou outros assuntos. Os processos foram acessados na íntegra, nos sites dos Tribunais Regionais Federais (TRF), a partir da sua numeração.

A coleta foi realizada de novembro de 2023 a outubro de 2024 por uma equipe de pesquisadores e consolidada em um banco de dados. As variáveis coletadas envolveram características dos demandantes, processuais, das prescrições e informações referentes aos custos dos produtos. Para esta análise, considerou-se apenas aquelas relacionadas a diagnósticos e idade dos demandantes.

Os diagnósticos e os respectivos códigos da 10ª revisão da Classificação Internacional de Doenças (CID-10) foram obtidos nas prescrições e relatórios médicos contidos nos processos. O formulário de coleta permitiu registrar até três diagnósticos por paciente.

### Grupos de diagnósticos e indicações

Os processos traziam, além do código CID-10, o nome relacionado ao mesmo e/ou uma descrição da condição do paciente. Foram delimitados grandes grupos de diagnósticos, sendo alguns compostos por apenas um código da CID-10, e outros por dois ou mais, conforme as características do diagnóstico, comorbidade ou região anatômica da condição clínica. Os grupos de diagnósticos receberam denominações que remetem às características das condições de saúde (Quadro 1). O grupo “outros” agregou diagnósticos primários de baixa frequência (menos de cinco ocorrências).

**Quadro 1:** Grupos de diagnósticos, código da 10ª revisão da Classificação Internacional de Doenças (CID-10) e descrições presentes nas demandas judiciais movidas contra a União, solicitando *Cannabis* medicinal. Brasil, 2020-2023.

GRUPO DE DIAGNÓSTICOS	CÓDIGO CID-10	DESCRIÇÃO
Deficiência intelectual	F70-F79	Transtornos do desenvolvimento intelectual que decorrem de déficit precoce no funcionamento cognitivo e no comportamento adaptativo
Demência	F00, F01, F02, F03, G30	Condições que envolvem deterioração de múltiplas funções cognitivas, como memória, raciocínio, linguagem e julgamento, sem perda da consciência, incluindo a doença de Alzheimer, doenças vasculares e outras condições neurológicas
Dor	D86, G43, G44, G50, G53, G62, M05, M14, M15, M17, M47, M50, M51, M54, M75, M86, M89, R51, R52	Fibromialgia, mialgia e dores inespecíficas, e/ou crônicas
Encefalopatia	G92, G93.4, G93.5, G93.6, G93.7, G93.8, G93.9, P91.6, P91.8, P91.9, P91.0	Encefalopatias com origens distintas: encefalopatia tóxica, encefalopatia não especificada, encefalopatias do recém-nascido, e outras doenças relacionadas ao encéfalo
Epilepsia	G40, G41, Q85.1	Condições neurológicas que implicam predisposição do cérebro para originar crises epilépticas. Englobam epilepsias, síndromes epiléticas e estados epiléticos, esclerose tuberosa em que há manifestações epiléticas
Esclerose múltipla	G35	Doença neurodegenerativa, imunomediada e desmielinizante que atinge o sistema nervoso central
Microcefalia	Q02	Malformação congênita do sistema nervoso que resulta em redução do perímetro cefálico
Neoplasia	C00-C97 e D00-D48	Todas as neoplasias, como neoplasias malignas, neoplasias *in situ*, neoplasias benignas e neoplasias de comportamento incerto ou desconhecido
Paralisia cerebral	G80	Transtorno neurológico de desenvolvimento caracterizado por uma lesão ou desenvolvimento anormal do cérebro que afeta o movimento, o tônus muscular e as funções motoras
Parkinson	G20	Mal de Parkinson, condição neurológica degenerativa que afeta principalmente o controle motor
Síndrome de Down	Q90	Todas as situações relacionadas à trissomia 21 e síndrome de Down não especificada
TDAH	F90	Transtorno hipercinético caracterizado por um padrão persistente de desatenção e/ou hiperatividade/impulsividade
TEA	F84	Transtornos globais do desenvolvimento, condições que abrangem o autismo infantil, a síndrome de Asperger e o autismo atípico
Ansiedade e depressão	F32, F33, F41	Transtornos relacionados à saúde mental, envolvendo ansiedade e/ou depressão
Outros	Vários	Diversas condições de saúde citadas em menos de cinco processos entre os consultados no estudo

TDAH: transtorno de déficit de atenção com hiperatividade; TEA: transtorno do espectro autista.

Fonte: elaboração própria, a partir de processos judiciais Departamento de Gestão das Demandas em Judicialização na Saúde (DJUD) do Ministério da Saúde.

A primeira análise descritiva das indicações foi realizada apenas para o diagnóstico primário, ou seja, aquele listado como principal no relatório médico de cada processo. Uma outra análise foi realizada considerando todos os diagnósticos - primários, secundários e terciários - para os mesmos pacientes. A distribuição de frequência dos diagnósticos primários e dos diagnósticos totais foi calculada.

Foi realizada ainda a análise cruzada, confrontando os diagnósticos e a idade dos pacientes demandantes da *Cannabis* medicinal. Para isso, os diagnósticos foram distribuídos de acordo com faixas etárias estabelecidas e foram construídos *heatmaps*. Essa análise buscou verificar a existência de algum padrão de utilização dos produtos frente ao perfil etário dos pacientes.

### Síntese qualitativa rápida de evidências

A seguir, foi realizada uma síntese qualitativa rápida de evidências (SQRE), método estabelecido na literatura ^13^ ^,^ ^14^, relacionada ao uso da *Cannabis* medicinal para as condições de saúde apontadas nos processos. A SQRE emprega passos definidos para sintetizar dados de pesquisa com maior rapidez que uma revisão rápida ou uma síntese qualitativa tradicional, produzindo um sumário baseado nas evidências mais metodologicamente rigorosas disponíveis e priorizando como fonte revisões sistemáticas, metanálises e revisões de revisões de alta qualidade, desde que incluam avaliação da certeza da evidência pelo *Grading of Recommendations Assessment, Development and Evaluation* (GRADE) ^15^ ou confiabilidade da mesma, julgada pelo nível de evidência e grau de recomendação da intervenção por outras ferramentas de avaliação ^16^ ^,^ ^17^ ^,^ ^18^ ^,^ ^19^. 

Para a SQRE, seguiu-se a recomendação: (i) delimitação da pergunta “existem evidências do uso de *Cannabis* medicinal (em suas variadas formas) para a indicação ‘X’?”; (ii) elegibilidade: revisões sistemáticas, revisões sistemáticas com metanálise e revisões de revisões de alta qualidade, sem delimitação de idioma e com delimitação temporal até dezembro de 2023, data limite dos processos; exclusões (duplicações, referências apenas com desfechos de segurança, farmacodinâmica ou farmacocinética da *Cannabis*, revisões sem avaliação da qualidade das evidências ou do nível de evidência por risco de viés/grau de recomendação e revisões sem acesso livre e gratuito); (iii) busca: partiu-se da Biblioteca Virtual em Saúde (BVS), em sua funcionalidade *Mapa de Evidências sobre Efetividade da Cannabis Medicinal* ^20^, da busca direta nas bases da Biblioteca Cochrane (https://www.cochranelibrary.com), e de funcionalidades de inteligência artificial generativa ^21^, usando a mesma pergunta delimitada pela SQRE. Todas as referências foram verificadas para certificação de fonte e autoria; (i) seleção: referências relacionadas aos grandes grupos de diagnósticos no estudo (Quadro 1); (ii) extração de dados: título, autores, ano de publicação, tipo de estudo, país, indicação clínica estudada, característica da tecnologia (princípio ativo ou produto estudado e eventuais comparadores - placebo ou outros medicamentos), resultados específicos de eficácia e/ou efetividade e conclusão do estudo; (iii) síntese: súmula qualitativa do agregado de informações relacionadas aos desfechos estudados, permitindo a denotação objetiva de uma de três possíveis conclusões para a prática clínica: (1) evidência de qualidade muito baixa ou baixa/de nível baixo, por alto risco de viés dos estudos/sem recomendação de emprego clínico, ou texto indicativo de não adoção na pratica clínica; (2) evidência de qualidade moderada-baixa baseada em poucos estudos de baixa qualidade metodológica ou inconclusiva, ou mesmo relato de falta de evidências ou de insuficiente número de estudos; e (3) evidências de qualidade alta, moderada-alta ou com nível alto, por baixo risco de viés/com recomendação de emprego clínico ou texto claramente favorável à intervenção.

### Trabalho da equipe e aprovação ética

A pesquisa envolveu nove pesquisadores de campo para coleta e extração de dados dos processos. Seis outros pesquisadores analisaram os dados e realizaram as demais etapas, trabalhando em duplas, de forma independente, na seleção e extração de dados da SQRE. 

O projeto foi aprovado pelo Comitê de Ética em Pesquisa da Escola Nacional de Saúde Pública Sergio Arouca, Fundação Oswaldo Cruz (ENSP/FIOCRUZ; CAAE 73301723.6.0000.5240).

## Resultados

### Análise das demandas

Foram analisadas 2.024 demandas judiciais referentes a PCM, ajuizadas contra o ente federal no período. Destas, 16,3% eram referentes ao ano de 2020, 25,79% ao ano de 2021, 34,14% ao ano de 2022 e 23,76% ao ano de 2023.

Com relação às características dos demandantes, 55,58% eram do sexo masculino, 56,62% eram menores de 14 anos e 6,57% tinham mais de 60 anos.

### Análise de diagnósticos e indicações

Para 62 processos, não havia informação sobre o diagnóstico do paciente. Foram identificados 1.962 diagnósticos primários e 3.757 diagnósticos totais (Tabela 1).

**Tabela 1:** Diagnósticos primários e totais, presentes nas demandas judiciais de produtos contendo *Cannabis* impetradas contra a União. Brasil, 2020-2023.

Diagnóstico	Primário	Total
n (%)	n (%)
Epilepsias	719 (36,65)	1.091 (29,04)
TEA	501 (25,54)	688 (18,31)
Dor	175 (8,92)	329 (8,54)
Paralisias cerebrais	142 (7,24)	310 (8,01)
Ansiedade e depressão	52 (2,65)	135 (3,57)
Deficiência intelectual	43 (2,19)	264 (7,00)
Demências	40 (2,04)	49 (1,30)
Parkinson	35 (1,78)	36 (0,96)
Microcefalia	16 (0,82)	68 (1,81)
TDAH	14 (0,71)	89 (2,37)
Neoplasias	14 (0,71)	20 (0,53)
Síndrome de Down	13 (0,66)	24 (0,64)
Esclerose múltipla	10 (0,51)	10 (0,27)
Encefalopatias	9 (0,46)	35 (0,90)
Outros	179 (9,12)	595 (16,74)
Total	1.962 (100,00)	3.757 (100,00)

TDAH: transtorno de déficit de atenção com hiperatividade; TEA: transtorno do espectro autista.

Fonte: elaboração própria.

A análise dos diagnósticos primários, ou seja, aqueles que impulsionaram a prescrição, revelou que a epilepsia (36,24%), o transtorno do espectro autista (TEA) (25,54%), a dor (8,92%) e a paralisia cerebral (7,24%) foram as condições mais frequentemente encontradas. Com relação ao total de diagnósticos, observou-se um padrão semelhante de frequência, mas com destaque para as condições de deficiência intelectual (7%) e transtorno de déficit de atenção com hiperatividade (TDAH) (2,37%), que surgiram com razão primário/total maior que a dos demais.

Já os diagnósticos presentes na categoria “outros” foram diversos e tiveram contribuição importante (16,74%) no total. No entanto, tiveram baixa frequência individual: síndrome de Ehlers-Danlos, transtorno desafiador e opositor (TOD), síndrome de Gilles de la Tourette, estresse pós-traumático, cisticercose, toxoplasmose congênita, asma, citomegalovírus, lúpus eritematoso sistêmico, psoríase, HIV, endometriose, incontinência fecal, hipertiroidismo, hipertensão arterial etc.

**Figura 1:**
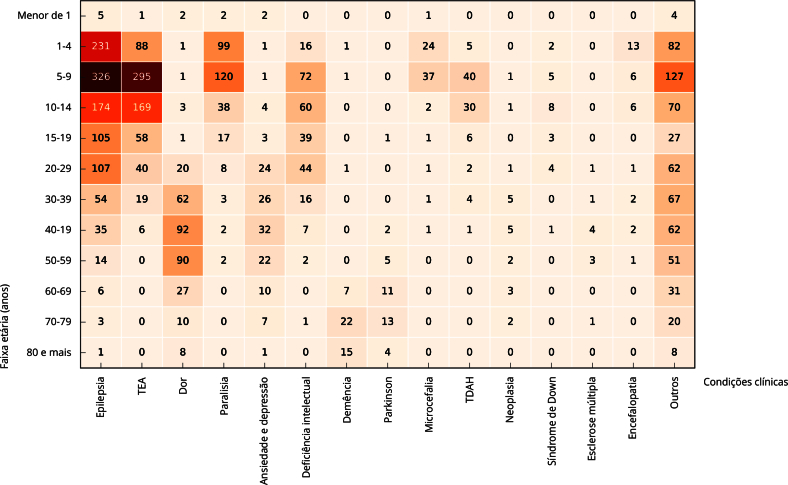
Diagnósticos primários presentes nas demandas judiciais de produtos contendo *Cannabis* impetradas contra a União, frente à idade dos pacientes demandantes. Brasil, 2020-2023.

TDAH: transtorno de déficit de atenção com hiperatividade; TEA: transtorno do espectro autista. Fonte: elaboração própria.

A análise das indicações frente à idade dos pacientes trouxe a configuração ilustrada pela Figura 1.

Expressiva parte dos diagnósticos foi oriunda de população de faixa etária de 1-4, 5-9, 10-14, e 15-19 anos, com epilepsia, TEA, paralisia e deficiência intelectual, seguida pelos adultos jovens (20-29 anos), com epilepsia, deficiência intelectual, ansiedade e depressão, e TEA. A categoria “outros” teve destaque nas faixas de crianças e jovens. A dor foi o grupo de diagnósticos mais prevalente entre adultos de 30-60 anos, seguido por ansiedade e depressão. Entre os idosos, as mais prevalentes foram dor, demência e Parkinson. 

### Síntese qualitativa rápida de evidências

Foram resgatadas 115 revisões que se ajustavam aos critérios de elegibilidade, detalhadas no Material Suplementar (https://cadernos.ensp.fiocruz.br/static//arquivo/supl-e00220525_6580.pdf).

A SQRE contemplou as indicações contidas nos processos (Tabela 1 e Figura 1). As 115 revisões foram publicadas entre 2011 e 2023, sendo os anos de 2021 e 2022 aqueles com maior número de publicações, 18,26% e 20%, respectivamente. Os países que mais publicaram foram os Estados Unidos (33,04%), o Reino Unido (18,26%) e o Chile (13,04%). Da base Cochrane vieram dez revisões (8,69%). Os diagnósticos mais estudados foram dor (40,86%), outros (32,17%), neoplasia (10,43%), epilepsia (9,56%) e ansiedade (3,47%). Houve grande diversidade de PCM empregados pelos estudos presentes nas revisões, como extratos, tinturas, óleos, canabinoides inespecíficos, canabinoides sintéticos, THC, CBD, canabinol (CBN), nabiximols e outras combinações CBD:THC, nabilona, dronabinol, folhas, planta integral (ingerida ou vaporizada e aspirada) e medicamento à base de *Cannabis* (Material Suplementar; https://cadernos.ensp.fiocruz.br/static//arquivo/supl-e00220525_6580.pdf). Uma análise pormenorizada desses produtos foge ao escopo deste artigo, sendo abordada em outro recorte.

**Figura 2:**
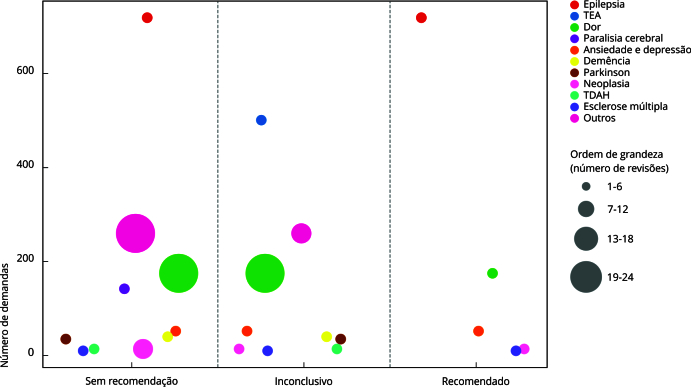
Condições clínicas relacionadas ao uso de *Cannabis* medicinal, segundo recomendação e número de demandas judiciais. Brasil, 2025.

TDAH: transtorno de déficit de atenção com hiperatividade; TEA: transtorno do espectro autista. Fonte: elaboração própria.

A Figura 2 mostra a relação entre grupos de diagnósticos e as recomendações para emprego clínico de PCM, oriundas da SQRE.

Para 1.962 diagnósticos primários presentes nos 2.024 processos (Tabela 1), duas condições clínicas - epilepsia e esclerose múltipla - apresentaram maior proporção de recomendação, face ao número de revisões (54,54% e 33,34%, respectivamente). Já para ansiedade e depressão, essa proporção foi de 25%; para neoplasia, 8,33% e para dor, 4,25%. A Figura 2 mostra que houve poucas revisões (representadas pelo diâmetro da esfera) para diagnósticos muito frequentes; e para diagnósticos com maior número de revisões não houve necessariamente recomendação resultante.

Em relação à epilepsia, foram encontradas 11 revisões que relataram como intervenção o CBD isolado. Sete revisões usaram avaliação de qualidade segundo GRADE. Três apontaram alta certeza de evidência para efetividade ^5^ ^,^ ^22^ ^,^ ^23^ e quatro, certeza de evidência moderada-baixa, baixa e muito baixa para emprego clínico. Das quatro revisões que usaram outras ferramentas, três fizeram recomendação de uso ^24^ ^,^ ^25^ ^,^ ^26^ e uma não recomendou o emprego clínico.

Para os efeitos espásticos na esclerose múltipla, foram encontradas nove revisões, seis das quais reportavam qualidade da evidência (GRADE). Entre essas, apenas uma ^27^ apresentou conclusões com alto grau de certeza, favorável ao uso da *Cannabis* em esclerose múltipla. As demais sustentam que canabinoides, especialmente nabiximols (THC:CBD em iguais proporções), oferecem benefício clínico modesto na espasticidade de adultos com esclerose múltipla refratária a terapias usuais, com certeza moderada de evidência. Das três com outras ferramentas de avaliação, duas não recomendavam o emprego clínico; a terceira ^28^, que considerou evidências do mundo real (estudos observacionais), recomendava.

Para ansiedade e depressão, foram quatro revisões. Duas reportaram avaliação da qualidade da evidência (GRADE), mas com certeza de evidência baixa e muito baixa. Duas usaram outras ferramentas, tendo uma recomendado o uso clínico ^29^ apenas para ansiedade; a outra não.

O uso de *Cannabis* em neoplasia aportou 12 revisões que relatavam o emprego para anorexia/caquexia, atividade antitumoral, êmese e sem especificação de sintoma ou atividade. Dez traziam a avaliação da qualidade (GRADE) e oito apontaram evidência baixa ou muito baixa para uso de PCM. Duas revisões tiveram recomendação inconclusiva. Para revisões que usaram outras ferramentas, uma apontou falta de recomendação e outra recomendou uso clínico para aumento de apetite ^30^.

O diagnóstico de dor foi frequente e agregou diferentes tipos e etiologias (aguda, crônica, ortopédica, orofacial, oncológica, do encéfalo, neuropática, reumatológica, psicossomática [fibromialgia] etc.). Nas 47 revisões que abordaram dor, 31 traziam avaliação da qualidade das evidências (GRADE). Quinze revisões tiveram desfechos inconclusivos, sem recomendação. Um só estudo ^5^ relatou evidência de qualidade alta para dor crônica em diferentes condições clínicas. Das 16 revisões que utilizaram outras ferramentas de avaliação, oito apontaram falta de recomendação e oito foram inconclusivas.

Para os diagnósticos relacionados ao TEA, só havia uma revisão com avaliação de qualidade (GRADE), com evidência moderada a baixa para emprego. A percepção de melhora de sintomas, como hiperatividade, automutilação, raiva, ansiedade, depressão, agressividade e diminuição do uso de medicamentos psicotrópicos e outros medicamentos não apresentou recomendação de emprego clínico conclusivo.

A paralisia cerebral engloba transtornos neurológicos de desenvolvimento e distúrbios motores. Duas revisões com ferramentas de avaliação (nenhuma com GRADE) concluíram que não há evidência de nível suficientemente alto que recomende o uso rotineiro de canabinoides para esses transtornos.

Na doença de Parkinson, o emprego da *Cannabis* controlaria os problemas motores. Foram cinco revisões, três com GRADE. Uma apontou a não redução dos sintomas ou discinesias da doença de Parkinson e as demais trouxeram resultados inconclusivos. Duas revisões que usaram outras ferramentas sugeriram não recomendação para a prática clínica.

Para demência, foram três revisões, sendo uma com GRADE, mas com insuficiência de certeza da evidência (evidência baixa ou muito baixa de efetividade) para recomendar o uso de canabinoides. As duas, com outras ferramentas de avaliação, não tiveram qualquer resultado conclusivo.

Para TDAH, foram encontradas duas revisões, ambas com avaliação da qualidade de evidência (GRADE) classificada como de baixa e muito baixa certeza. Considerando as evidências encontradas, não se pode recomendar o uso de canabinoides para tratamento de TDAH.

Para o grupo outros, foram encontradas 37 revisões. Essas condições são múltiplas: esquizofrenia, HIV, colite ulcerativa, glaucoma, síndrome de Tourette, estresse pós-traumático, cuidados paliativos, sono, lesões medulares etc. (Material Suplementar; https://cadernos.ensp.fiocruz.br/static//arquivo/supl-e00220525_6580.pdf). Destas, 23 tinham avaliação da qualidade da evidência (GRADE), mas nenhuma delas com alta certeza, e, portanto, sem suporte conclusivo para recomendação clínica. Das 14 revisões avaliadas por outras ferramentas, nenhuma delas foi conclusiva pela recomendação clínica de uso em quaisquer das indicações, sendo oito com recomendação contrária ao uso clínico. Seis destacaram a incerteza das evidências produzidas, sua falta de qualidade ou resultados modestos ou inconclusivos.

Para outros grupos de diagnósticos específicos presentes nos processos (Tabela 1), como síndrome de Down, microcefalia (qualquer etiologia), deficiência intelectual e encefalopatia, não foram resgatadas revisões publicadas sobre uso terapêutico de *Cannabis*/canabinoides até dezembro de 2023.

## Discussão

Nas 2.024 demandas judiciais que pleiteavam PCM no período analisado, identificaram-se 15 grandes grupos diagnósticos. Observou-se concentração em 2.022, possivelmente relacionada à publicação da *Resolução da Diretoria Colegiada RDC nº 660/2022* ^31^, que simplificou a importação de PCM. Predominaram crianças e adultos jovens com epilepsia, TEA, paralisia e deficiência intelectual entre os demandantes. Os achados convergem com dois estudos ^32^ ^,^ ^33^ que trataram do uso de PCM no cenário da judicialização, ambos analisando notas técnicas dos Núcleos de Apoio Técnico do Judiciário (NatJus), e que também destacaram epilepsia, TEA, dor e paralisia.

As revisões disponíveis eram oriundas dos Estados Unidos e do Reino Unido em sua maioria, sendo uma porcentagem expressiva do Chile. Para os dois primeiros, a maior produção é reflexo dos contextos regulatórios que fomentam a pesquisa ^34^. Já o Chile é um dos países da América Latina que mais cedo aceitou o uso de PCM pela chamada legalização de fato, uma flexibilização do arcabouço regulatório ^35^.

A SQRE estimou a certeza embutida nas prescrições demandando PCM. Uma revisão sistemática e outras revisões de alta qualidade levam, em média, dois anos para conclusão ^36^, com pergunta bem definida, ajudando a garantir qualidade, porém limitando o escopo. Por outro lado, a SQRE parte de revisões concluídas que utilizaram ferramentas para avaliação de qualidade, oferecendo síntese ágil para embasar decisões clínicas e de gestão ^37^.

Tem havido um crescimento acelerado de clínicas e prescritores que praticam a “medicina canábica” em vários países, inclusive no Brasil ^32^, com prescrições de PCM individualizadas para diversos diagnósticos ^38^. Em âmbito privado, desde que guardada a observância ética e legal, as escolhas terapêuticas são admitidas - ainda que potencialmente inapropriadas. Na presente análise, contudo, a maioria das demandas careceu de evidências com certeza suficiente para sustentar as indicações. Considerando que a síntese abrange estudos até 2023, seria razoável esperar que os prescritores já tivessem acesso às mesmas evidências ao indicar ou solicitar PCM.

Somente cinco condições - epilepsia, esclerose múltipla, ansiedade, neoplasia e dor - apresentaram algum suporte na literatura, em graus diferentes e com baixa frequência de revisões de alta qualidade (Figura 2). Das condições citadas, a epilepsia se destaca por trazer revisões em que a intervenção testada foi o CBD, direcionando as conclusões de forma mais indicativa ao seu uso clínico. Na esclerose múltipla, o uso de nabiximols trouxe revisões com alguma certeza de evidência. Este produto é o único medicamento à base de *Cannabis* no mercado ^39^. Para ser registrado, o fabricante teve de submeter estudos clínicos comprovando eficácia em espasticidade, notadamente no contexto da esclerose múltipla ^40^. 

Dor foi o desfecho com maior número de revisões, em geral focadas nos efeitos dos PCM sobre a dor preexistente, e com achados majoritariamente inconclusivos ou de não recomendação. Os efeitos dos diversos PCM sobre o sistema endocanabinoide ainda estão sendo elucidados. Há trabalhos que hipotetizam que, para além dos variados tipos de dor e dos confundimentos potenciais dos estudos, a ação possível dos PCM seria sobre o limiar da dor, e não sobre a dor já instalada ^41^, ou mesmo similar ao efeito placebo ^42^. Isso subverteria as formas de investigação e de mensuração de desfechos, o que poderia explicar o insucesso das pesquisas em revelar resultados de efetividade.

A literatura produzida a partir de janeiro de 2024 até outubro de 2025 não parece modificar o panorama observado na SQRE. Entre novas revisões com avaliação de qualidade: para epilepsia, a certeza de evidência foi muito baixa/baixa para emprego clínico ^43^; para TEA, os efeitos permaneceram inconclusivos ^44^; na espasticidade em esclerose múltipla, uma revisão trouxe benefícios definidos ^45^, enquanto duas o relativizaram pela alta heterogeneidade e vieses dos estudos ^46^ ^,^ ^47^; para dor, incluindo fibromialgia ^47^ ^,^ ^48^, as revisões trouxeram benefícios pequenos, individualizados e de curto prazo, com certeza de evidência moderada a baixa; para náuseas em neoplasia ^49^, houve não recomendação de uso; e em demência ^50^, não surgiram novas evidências que substanciassem o uso clínico.

Diversas revisões analisadas apontaram, recorrentemente, baixa qualidade de estudos primários - falhas de cegamento, desenho, heterogeneidade das intervenções e mensuração de desfechos/exposições - o que dificulta detectar efeitos e comparar resultados. Soma-se a isso o uso de amostras pequenas e de estudos observacionais frágeis como potenciais fontes de “evidências” ^51^ ^,^ ^52^ ^,^ ^53^, em meio ao crescente apelo por dados do mundo real, para formação de evidências de mundo real em tratamentos com *Cannabis* ^54^. No entanto, estudos clínicos controlados de qualidade - considerados o padrão-ouro para recomendação de uso - ainda são poucos, não se justificando o emprego intenso, variado e não regulamentado de substâncias que nem sempre detêm o status de medicamento com base em estudos observacionais. O movimento de expansão do uso de PCM cumpre agenda múltipla, que pode estar tendo o efeito de medicalizar o uso da *Cannabis*, apresentando-a como solução para variados problemas cotidianos ^55^, fenômeno possivelmente reforçado por matérias de mídia que superlativizam seus efeitos em doenças comuns ^7^. Em algumas indicações, é plausível que a prescrição esteja sendo guiada mais pela percepção de benefícios do que por evidências robustas ^56^ ^,^ ^57^. 

Diagnósticos com volume muito elevado de demandas, como TEA (25,5% do total), não apresentaram evidências que sustentassem a prescrição. Se fossem medicamentos, estariam abordados pelos *Temas* 1.234 ^58^ e 6 ^59^ do Supremo Tribunal Federal, exigindo apreciação baseada em evidências robustas; como PCM não são medicamentos, a legitimidade do fornecimento público deve ser questionada. Cabe, em última análise, estimar não apenas se havia evidências suficientemente robustas que recomendariam o emprego clínico, mas também se justificaria o fornecimento público desses produtos, com ônus financeiro sobre a sociedade. 

Apesar disso, muitos estados e municípios vêm incluindo PCM em suas listas de dispensação ^60^ ^,^ ^61^, sem reflexão que se sustente para além das simples responsabilidades de pagamento. Adotar PCM sem evidências para evitar despesas de judicialização é questionável em termos de gestão sanitária. Ademais, a expectativa de que o ente federal assuma a despesa não se sustenta: além de não “incorporados” pela Conitec, por falta de evidências, os PCM tampouco são categorizados como medicamentos pela Anvisa ^12^.

Preocupa a predominância de crianças e adolescentes como demandantes. Os dados de segurança dos PCM ainda são limitados, assim como os efeitos potenciais sobre as condições médicas subjacentes e as interações medicamentosas, face à variedade de tipos e origens desses produtos, tecnicamente não padronizados ou regulamentados ^62^. A maioria das revisões avaliou exposições de curto prazo (Material Suplementar; https://cadernos.ensp.fiocruz.br/static//arquivo/supl-e00220525_6580.pdf); o perfil de segurança em uso diário prolongado, tanto do CBD como de outros PCM, permanece amplamente desconhecido ^63^.

A percepção de que há poucos estudos sobre os efeitos de PCM não se concretizou pela SQRE. Havia um número expressivo de revisões disponíveis; por outro lado, a ampla variedade de indicações e formas de uso leva muitas revisões a mesclar estudos com planta integral (fumada, ingerida, vaporizada/inalada, adesivos transdérmicos) e com derivados isolados ou em combinações (particularmente o THC e o CBD), o que torna o processo de produção de evidências pouco sistemático. Essa heterogeneidade dinamiza o mercado, mas pouco orienta o desenvolvimento de medicamentos e ganhos clínicos tangíveis. Não surpreende, portanto, que várias revisões permaneçam inconclusivas e reforcem a necessidade de estudos adicionais.

Entre as limitações, ressalta-se que as indicações analisadas provêm das demandas judiciais que solicitam fornecimento dos produtos pelo Estado e, portanto, podem não refletir a totalidade do uso da *Cannabis* medicinal no Brasil. Entretanto, por abranger quatro anos e todas as Unidades da Federação, acredita-se que esse recorte sirva como *proxy* do cenário de utilização de PCM no país.

A SQRE foi utilizada especificamente para avaliar criticamente a força e as limitações das evidências disponíveis, e não avaliar processos. Privilegia rigor metodológico, transparência, rastreabilidade e utilidade prática, oferecendo súmula oportuna. A literatura indica alta congruência entre revisões sistemáticas e sínteses rápidas de evidências ^64^ ^,^ ^65^. Foram incluídas apenas revisões de acesso público e completo, potencialmente restringindo resultados, porém reforçando a garantia de que aquelas disponíveis o seriam para todos os envolvidos na tomada de decisão.

Há relatos na literatura de ganhos terapêuticos em contextos de uso compassivo de cuidados paliativos e ganhos individuais que não geram evidências. De fato, o estudo não foi capaz, ou mesmo teve a intenção, de captar essas situações, uma vez que o foco foram as evidências que possam justificar a oferta pública.

Por fim, parte dos diagnósticos listados nos processos pode estar confundidos com o controle dos sintomas, potencial alvo clínico das demandas por PCM, em detrimento das condições clínicas em si. Entretanto, seria impossível estimar a real intenção dos prescritores.

O Estado, cumprindo seu papel de decisor sobre o fornecimento público de produtos para a saúde, deve se manifestar inequivocamente quanto à *Cannabis* medicinal. É preciso aperfeiçoar e tornar explícita a regulação dos PCM ^12^ ^,^ ^66^, oferecendo base regulatória responsável que defina, além das responsabilidades de financiamento, o que é aceitável em termos de proteção da saúde pública.

## Conclusão

O estudo analisou demandas judiciais contra a União que pleiteavam *Cannabis* medicinal, confrontando os diagnósticos contidos nos processos com uma SQRE. Essa, por sua vez, se propôs a sintetizar evidências já consolidadas e com qualidade avaliada, de modo a assegurar parâmetro para a análise.

Entre 2020 e 2023, os processos trouxeram poucas indicações com evidências suficientemente robustas. Essas conclusões não visam a julgar o mérito das ações, mas se as prescrições que as embasaram estavam inequivocamente baseadas em evidências científicas, critério essencial para legitimar o fornecimento público.

A casuística dos processos indica predominância de crianças e adolescentes entre os demandantes expostos a PCM variados - produtos sem avaliação de qualidade - e cerca de metade das indicações se apoia em fraca ou nenhuma evidência, o que causa preocupação.

A grande quantidade de indicações sem evidências e a pressão da judicialização configuram risco sanitário considerável que não tem tido abordagem tempestiva no país. É urgente regulamentar os PCM e sua prescrição. A proteção sanitária deve se efetivar para todas as prescrições e pacientes. No entanto, às prescrições que se efetivam pelo fornecimento público obriga-se amparo em evidências robustas.

## Data Availability

As fontes de informação utilizadas no estudo estão indicadas no corpo do artigo.

## Material Suplementar

Material Suplementar
