# Supplementary material for: Therapeutic indications in lawsuits seeking medical Cannabis in Brazil: a cross-sectional study in light of scientific evidence
Source: Cad Saude Publica. 2026 Jul 6;42:e00220525. [Article in Portuguese] doi: 10.1590/0102-311XPT220525 (PMC13336621; doi:10.1590/0102-311XPT220525)
Supplement: Material Suplementar [file 1678-4464-csp-42-PT220525-s.pdf]

Material Suplementar

Súmula de evidências sobre produtos de cannabis medicinal. Resultado da Síntese Qualitativa Rápida de Evidências (SQRE) realizada para as indicações terapêuticas contidas em demandas judiciais que pleiteiam cannabis medicinal. Brasil, 2025.

Autor(res)	Ano de publicação	País de publicação	Tipo de estudo	Título	Ferramenta de avaliação	Síntese da evidência	Grupo de diagnósticos	Conclusões para a prática clínica
Lynch ME, Campbell F.	2011	Inglaterra	Revisão Sistemática	Cannabinoids for treatment of chronic non-cancer pain; a systematic review of randomized trials	Oxford Quality score ou Jadad scale	A revisão sistemática demonstra que os canabinoides são uma opção de tratamento modestamente eficaz para dor crônica não oncológica (predominantemente neuropática). São necessários mais estudos de larga escala e de longo prazo.	Dor	2
Lutge EE, Gray A, Siegfried N.	2013	Cochrane Database	Revisão	The medical use of cannabis for reducing morbidity and mortality in patients with HIV/AIDS	Rob Tools (Cochrane)	Faltam evidências da eficácia da *Cannabis* e dos canabinoides na anorexia associada à AIDS. Os efeitos não estão claros. Os estudos realizados foram de curta duração, com um pequeno número de pacientes, e se concentraram em medidas de eficácia de curto prazo.	Outras	1
Gloss D, Vickrey B.	2014	Cochrane Database	Revisão Sistemática	Cannabinoids for epilepsy	RoB Tools (Cochrane)	No momento não se pode chegar a nenhuma conclusão confiável em relação à eficácia de uso de canabinoides no	Epilepsia	1

						tratamento da epilepsia. São necessários ensaios com maior número de pacientes.		
Andreae MH, Carter GM, Shaparin N, *et al.*	2015	Estados Unidos	Meta-análise	Inhaled cannabis for chronic neuropathic pain: an individual patient data meta-analysis	Rob Tools (Cochrane)	A meta-análise de dados individuais de pacientes sugere que a *Cannabis* inalada resulta em benefícios de curto prazo para dor neuropática crônica. Não há evidências sobre benefícios e riscos sustentados a longo prazo no ambiente comunitário, pelo alto risco de viés dos estudos.	Dor	2
Deshpande A, Mailis-Gagnon A, Zoheiry N, Lakha SF.	2015	Canadá	Revisão Sistemática	Efficacy and adverse effects of medical marijuana for chronic noncancer pain: Systematic review of randomized controlled trials	Oxford Quality score ou Jadad scale	Há evidências do uso de maconha medicinal em baixas doses para dor neuropática refratária em conjunto com analgésicos tradicionais. No entanto, os ensaios foram limitados pela curta duração, pela variabilidade na dosagem e na potência do delta-9-tetraidrocanabinol e pela falta de resultados funcionais. A generalização do uso de maconha medicinal para todas as condições de dor crônica não oncológica (DCNC)	Dor	2

						não parece ser apoiada pelas evidências existentes.		
Smith LA, Azariah F, Lavender VT, *et al.*	2015	Cochrane Database	Revisão	Cannabinoids for nausea and vomiting in adults with cancer receiving chemotherapy	RoB Tools (Cochrane)	A qualidade da evidência para a maioria dos desfechos é baixa. Questões metodológicas dos estudos limitaram as conclusões e outros futuros estudos sobre quimioterapia e ação antiemética podem modificar as conclusões.	Neoplasias	1
Fitzcharles MA, Ste-Marie PA, Häuser W, *et al.*	2016	Estados Unidos	Revisão Sistemática	Efficacy, Tolerability and Safety of Cannabinoid Treatments in the Rheumatic Diseases: A Systematic Review of Randomized Controlled Trials	GRADE	Atualmente, não há evidências suficientes para recomendar tratamentos com canabinoides para o manejo de doenças reumáticas, em função de amostras pequenas, tempo curto, heterogeneidade de condições e produtos e falta de estudos para produtos da erva *in natura*.	Dor	1
García K, Rada G.	2016	Chile	Revisão	Do cannabinoids have a role to play in Tourette's syndrome?	GRADE	A conclusão é de que não está claro se canabinoides reduzem tics em Síndrome de Tourette.	Outras	1
Lobos Urbina D, Peña Durán J.	2016	Chile	Revisão	Are cannabinoids effective for treatment of pain in patients with active cancer?	GRADE	A certeza da evidência é muito baixa, razão pela qual não está claro se canabinoides diminuem dor e	Neoplasias	1

						melhoram qualidade de vida em pacientes com câncer.		
Walitt B, Klose P, Fitzcharles MA, Phillips T, Häuser W.	2016	Cochrane Database	Revisão	Cannabinoids for fibromyalgia	GRADE	Qualidade da evidência é muito baixa em função de resulatdos indiretos, imprecisos e potenciais viéses para sugerir tratamento de fibromialgia com nabilona.	Dor	1
Whiting PF, Wolff RF, Deshpande S, *et al.*	2016	Estados Unidos	Revisão Sistemática e meta-análise	Cannabinoids for Medical Use: A Systematic Review and Meta-analysis	GRADE	Houve evidências de qualidade moderada para apoiar o uso de canabinoides no tratamento de dor crônica e espasticidade. Houve evidências de baixa qualidade sugerindo que os canabinoides foram associados à melhora de náuseas e vômitos devido à quimioterapia, ganho de peso na infecção pelo HIV, distúrbios do sono e síndrome de Tourette.	Dor<br>Neoplasias<br>Outras	2<br>1<br>1
Wilkinson ST, Radhakrishnan R, D'Souza DC.	2016	Estados Unidos	Revisão Sistemática	A Systematic Review of the Evidence for Medical Marijuana in Psychiatric Indications	GRADE	A força da evidência para uso de canabinoides nessas condições clínicas (Síndrome de Tourette, Síndrome de Estresse Pós-Traumático - SEPT, doença de Alzheimer), nesse momento, é muito baixa.	Outras	1
Javier Peña, Gabriel Rada	2017	Chile	Revisão	Are cannabinoids	GRADE	Não está claro se	Epilepsia	1

				effective for epilepsy?		canabinoides diminuem a frequência de convulsões em epilepsia, pois a certeza da evidência é muito baixa.		
Allende-Salazar RF, Rada G.	2017	Chile	Revisão	Are cannabinoids an effective treatment for chronic non-cancer pain?	GRADE	Não está claro se canabinoides diminuem dor em pacientes com dor crônica não oncológica, pois a certeza da evidência é muito baixa.	Dor	1
Amato L, Minozzi S, Mitrova Z, *et al.*	2017	Itália	Revisão Sistemática	Systematic review of safeness and therapeutic efficacy of cannabis in patients with multiple sclerosis, neuropathic pain, and in oncological patients treated with chemotherapy	GRADE	Em pacientes com esclerose múltipla, a confiança na estimativa foi alta em favor da *Cannabis* para espasticidade e dor. Já para dor crônica e neuropática, houve evidência de um pequeno efeito, mas com baixa confiança na estimativa. Há incerteza se a *cannabis* reduz náuseas e vômitos em pacientes com câncer que requerem quimioterapia, com confiança baixa ou muito baixa.	Esclerose Múltipla<br>Dor<br>Neoplasia	3<br>2<br>1
Aviram J, Samuelly-Leichtag G.	2017	Estados Unidos	Revisão Sistemática e meta-análise	Efficacy of Cannabis-Based Medicines for Pain Management: A Systematic Review and Meta-Analysis of Randomized Controlled Trials	Oxford Quality score ou Jadad scale	A revisão sistemática atual sugere que medicamentos à base de *Cannabis* podem ser eficazes no tratamento da dor crônica, com base em evidências limitadas,	Dor	2

						principalmente para pacientes com dor neuropática (DN).		
Bravo-Soto GA, Juri C.	2017	Chile	Revisão	Are cannabinoids effective for Parkinson's disease?	GRADE	Os canabinoides provavelmente não diminuem sintomas de Parkinson ou discinesia, com certeza moderada da evidência.	Parkinson	1
Cabeza C, Corsi O, Pérez-Cruz P.	2017	Chile	Revisão	Are cannabinoids an alternative for cachexia-anorexia syndrome in patients with advanced cancer?	GRADE	Não está claro se canabinoides possuem algum efeito positivo no ganho de peso, pois a certeza da evidência é muito baixa.	Neoplasias	1
Contreras T, Bravo-Soto GA, Rada G.	2017	Chile	Revisão	Do cannabinoids constitute a therapeutic alternative for anorexia nervosa?	GRADE	Não está claro se canabinoides aumentam ganho de peso ou reduzem sintomas na anorexia nervosa, pois a certeza da evidência é baixa.	Outras	1
Da Rovare VP, Magalhães GPA, Jardini GDA, *et al.*	2017	Escócia	Revisão Sistemática e meta-análise	Cannabinoids for spasticity due to multiple sclerosis or paraplegia:A systematic review and meta-analysis of randomized clinical trials	GRADE	Há evidências de certeza moderada quanto ao impacto dos canabinoides na espasticidade devido à esclerose múltipla ou paraplegia. São necessários mais e maiores estudos.	Esclerose múltipla	2
Guinguis R, Ruiz MI, Rada G.	2017	Chile	Revisão Sistemática	Is cannabidiol an effective treatment for schizophrenia?	GRADE	O canabidiol provavelmente não reduz os sintomas da esquizofrenia e não melhora os déficits cognitivos da esquizofrenia, com certeza da evidência moderada.	Outras	1
Häuser W, Fitzcharles MA, Radbruch L, Petzke F.	2017	Alemanha	Revisão Sistemática	Cannabinoids in Pain Management	AMSTAR ou AMSTAR-2	A percepção pública sobre a eficácia,	Dor	2

				and Palliative Medicine		tolerabilidade e segurança dos medicamentos à base de *Cannabis* no tratamento da dor e na medicina paliativa conflita com os resultados de revisões sistemáticas e estudos observacionais prospectivos conduzidos de acordo com os padrões da medicina baseada em evidências. Concluiu-se que os dados são inadequados para apoiar ou refutar o uso da maconha medicinal.		
Lim K, See YM, Lee J.	2017	Coreia do Sul	Revisão Sistemática	A Systematic Review of the Effectiveness of Medical Cannabis for Psychiatric, Movement and Neurodegenerative Disorders	Rob Tools (Cochrane)	Embora existam ensaios clínicos que sugerem o potencial benefício dos canabinoides para anorexia nervosa, ansiedade, SEPT, sintomas psicóticos de agitação na doença de Alzheimer e demência, doença de Huntington e síndrome de Tourette, e discinesia na doença de Parkinson, conclusões insuficientes podem ser tiradas devido à baixa qualidade das evidências,	Outras	1

						conforme indexado pelo risco de viés Cochrane, e amostras com poder estatístico insuficiente.		
Meng H, Johnston B, Englesakis M, *et al.*	2017	Estados Unidos	Revisão Sistemática e meta-análise	Selective Cannabinoids for Chronic Neuropathic Pain: A Systematic Review and Meta-analysis	GRADE	Canabinoides seletivos proporcionam um pequeno benefício analgésico em pacientes com dor neuropática crônica. Houve um alto grau de heterogeneidade entre as publicações incluídas neste SR-MA. São necessários estudos grandes de maior duração e com desenho metodológico randomizado para melhor avaliação.	Dor	2
Meza R, Peña J, García K, *et al.*	2017	Chile	Revisão	Are cannabinoids effective in multiple sclerosis?	GRADE	O uso de canabinoides em esclerose múltipla não reduz a espasticidade ou a dor, com alta certeza de evidência.	Esclerose múltipla	1
Morales M, Corsi O, Peña J.	2017	Chile	Revisão	Are cannabinoids effective for the management of chemotherapy induced nausea and vomiting?	GRADE	Não está claro se canabinoides beneficiam pacientes com náuseas e vômitos em quimioterapia, pois a certeza da evidência é muito baixa.	Neoplasias	1
Núñez A, Núñez C, Corsi O, Rada G.	2017	Chile	Revisão	Are cannabinoids effective for HIV wasting syndrome?	GRADE	Não está claro se canabinoides aumentam o apetite ou ganho de peso em pacientes com	Outras	1

						caquexia secundária ao HIV, pois a certeza da evidência é muito baixa.		
O'Neil ME, Nugent SM, Morasco BJ, *et al.*	2017	Estados Unidos	Revisão Sistemática	Benefits and Harms of Plant-Based Cannabis for Posttraumatic Stress Disorder A Systematic Review	Outra ferramenta	As evidências são insuficientes para tirar conclusões sobre os benefícios e malefícios das preparações de *Cannabis* à base da planta em pacientes com SEPT.	Outras	1
Stevens AJ, Higgins MD.	2017	Inglaterra	Revisão Sistemática	A systematic review of the analgesic efficacy of cannabinoid medications in the management of acute pain	GRADE	A revisão sistemática concluiu que, com base nas evidências disponíveis em ensaios clínicos randomizados controlados, os canabinoides não desempenham nenhum papel no tratamento da dor aguda.	Dor	1
Contreras T, Bravo-Soto G, Rada G.	2018	Chile	Revisão	Do cannabinoids constitute a therapeutic alternative for insomnia?	GRADE	Não está claro se os canabinoides afetam a gravidade da insônia ou qualidade do sono, pois a certeza das evidências é muito baixa. Não está claro se os canabinoides afetam o início do sono, pois a certeza das evidências é baixa.	Outras	1
De Vita MJ, Moskal D, Maisto SA, Ansell EB.	2018	Estados Unidos	Revisão Sistemática e meta-análise	Association of Cannabinoid Administration with Experimental Pain in Healthy Adults: A Systematic Review and Meta-analysis	GRADE	Os canabinoides podem prevenir o aparecimento da dor, produzindo pequenos aumentos nos limiares de dor, mas podem não	Dor	2

						reduzir a intensidade da dor experimental já vivenciada; em vez disso, os canabinoides podem tornar a dor experimental menos desagradável e mais tolerável, sugerindo uma influência nos processos afetivos.		
Pereira FA, Torres AC, Philadelpho VO, *et al.*	2018	Brasil	Revisão Sistemática	Efeitos do Canabidiol na frequência das crises epilépticas: uma revisão sistemática	CONSORT	O uso terapêutico do canabidiol foi associado à redução na frequência de crises epilépticas, além de melhoria global da qualidade de vida em indivíduos com epilepsia refratária. No entanto, novos estudos randomizados duplo-cego são necessários para validação interna e externa.	Epilepsia	3
Kafil TS, Nguyen TM, MacDonald JK, Chande N.	2018	Cochrane Database	Revisão	Cannabis for the treatment of ulcerative colitis	GRADE	Não está claro se os canabinoides apresentam algum benefício na colite ulcerativa, pois a certeza das evidências é baixa.	Outras	1
Mücke M, Phillips T, Radbruch L, *et al.*	2018	Cochrane Database	Revisão	Cannabis-based medicines for chronic neuropathic pain in adults	GRADE	Não está claro se os canabinoides apresentam algum benefício na dor neuropática crônica, pois a certeza das evidências é muito baixa. A qualidade das evidências para os desfechos de alívio da dor reflete	Dor	1

						a exclusão de participantes com histórico de abuso de substâncias e outras comorbidades significativas dos estudos, juntamente com o pequeno tamanho das amostras.		
Mücke M, Weier M, Carter C, *et al.*	2018	Alemanha	Revisão Sistemática e meta-análise	Systematic review and meta-analysis of cannabinoids in palliative medicine	GRADE	Não está claro se os canabinoides apresentam algum benefício em alterações no ganho de peso, ingestão alimentar (apetite) e náuseas em pacientes com câncer, pois a certeza das evidências é muito baixa.	Neoplasias	1
Peña J, Jiménez C, Schmidt J.	2018	Chile	Revisão	¿Tienen algún rol los cannabinoides en el control del glaucoma?	GRADE	Não está claro se canabinoides para glaucoma causa diminuição transitória da pressão intraocular, pois a certeza da evidência é muito baixa.	Outras	1
Rocco M, Rada G.	2018	Chile	Revisão	Are cannabinoids effective for fibromyalgia?	GRADE	Não está claro se os canabinoides apresentam algum benefício na fibromialgia, pois a certeza das evidências é muito baixa.	Dor	1
Rodríguez A, Zavala C.	2018	Chile	Revisão	Cannabinoids for the treatment of cannabis abuse disorder	GRADE	Não está claro se os canabinoides apresentam algum benefício no tratamento de abuso de *Cannabis*, pois a	Outras	1

						certeza das evidências é baixa.		
Stockings E, Campbell G, Hall WD, *et al.*	2018	Estados Unidos	Revisão Sistemática e meta-análise	Cannabis and cannabinoids for the treatment of people with chronic noncancer pain conditions: a systematic review and meta-analysis of controlled and observational studies	GRADE	Parece improvável que os canabinoides sejam medicamentos altamente eficazes para a dor crônica não oncológica (DCNO). Há evidências de nível moderado a alto que apoiam o uso de nabiximols para alcançar reduções modestas da dor como terapia adjuvante para a dor relacionada à EM. Ha evidências mínimas que apoiem efetividade de canabinoides em ou outros domínios importantes em pessoas com DCNO, como o funcionamento emocional e físico.	Dor<br>Esclerose Multipla	1<br>2
Stockings E, Zagic D, Campbell G, *et al.*	2018	Inglaterra	Revisão Sistemática	Evidence for cannabis and cannabinoids for epilepsy: a systematic review of controlled and observational evidence	GRADE	Uma proporção razoável de pacientes apresentou redução na frequência de convulsões ao usar produtos de CBD de grau farmacêutico em adição a antiepilépticos (DAEs), porém a certeza da evidência é baixa. São necessários mais estudos para estabelecimento de base que avalie riscos e benefícios	Epilepsia	2

						de canabinoides na epilepsia.		
Torres-Moreno MC, Papaseit E, Torrens M, Farré M.	2018	Estados Unidos	Revisão Sistemática e meta-análise	Assessment of Efficacy and Tolerability of Medicinal Cannabinoids in Patients with Multiple Sclerosis a Systematic Review and Meta-analysis	Rob Tools (Cochrane)	Os canabinoides produzem uma redução limitada e leve da espasticidade subjetiva, da dor e da disfunção da bexiga em pacientes com esclerose múltipla (EM), mas não alteram a espasticidade medida objetivamente.	Esclerose múltipla	1
Akgün K, Essner U, Seydel C, Ziemssen T.	2019	Estados Unidos	Revisão Sistemática	Daily Practice Managing Resistant Multiple Sclerosis Spasticity with Delta-9-Tetrahydrocannabinol: Cannabidiol Oromucosal Spray: A Systematic Review of Observational Studies	Outra ferramenta	Na espasticidade resistente à terapia o uso adicional de THC:CBD é uma opção terapêutica eficaz com um bom perfil nas primeiras quatro semanas de tratamento, de acordo com evidências de mundo real, proporcionada por estudos observacionais.	Esclerose múltipla	3
Bahji A, Meyyappan AC, Hawken ER.	2020	Estados Unidos	Revisão Sistemática e meta-análise	Cannabinoids for the Neuropsychiatric Symptoms of Dementia: A Systematic Review and Meta-Analysis	Rob Tools (Cochrane)	A revisão sistemática e meta-análise encontrou evidências consistentes de que os canabinoides são eficazes para o tratamento de sintomas neuropsiquiátricos (SNP) associados à demência, porém as evidências não foram robustas e foram	Demências	2

						particularmente vulneráveis aos pequenos tamanhos de amostra. São necessários mais estudos de base populacional para caracterizar efetividade e recomendar emprego clínico.		
Black N, Stockings E, Campbell G, *et al.*	2019	Inglaterra	Revisão Sistemática e meta-análise	Cannabinoids for the treatment of mental disorders and symptoms of mental disorders: a systematic review and meta-analysis	GRADE	Não está claro se os canabinoides apresentam algum benefício em transtornos e sintomas depressivos, transtornos de ansiedade, transtorno de déficit de atenção e hiperatividade, síndrome de Tourette, transtorno de estresse pós-traumático ou psicose, pois a certeza das evidências é muito baixa. São necessários mais estudos de alta qualidade que examinem diretamente o efeito dos canabinoides no tratamento de transtornos mentais.	Ansiedade e Depressão	1
							TDAH	1
							Outros	1
Häuser W, Welsch P, Klose P, *et al.*	2019	Alemanha	Revisão Sistemática e meta-análise	Efficacy, tolerability and safety of cannabis-based medicines for cancer pain: A systematic review with meta-analysis of	GRADE	Evidências de qualidade muito baixa sugerem que nabiximols oromucosais e THC não têm efeito sobre a dor, problemas de	Dor	1

				randomised controlled trials		sono e consumo de opioides em pacientes com dor oncológica com alívio insuficiente da dor por opioides.		
Hillen JB, Soulsby N, Alderman C, Caughey GE.	2019	Inglaterra	Revisão Sistemática	Safety and effectiveness of cannabinoids for the treatment of neuropsychiatric symptoms in dementia: a systematic review	Outra ferramenta	Embora a eficácia dos canabinoides não tenha sido comprovada em um ensaio clínico randomizado robusto, estudos observacionais mostraram resultados promissores, especialmente para pacientes com sintomas refratários. Mais estudos com exame de outras variáveis são necessários.	Demências	2
Hoch E, Niemann D, von Keller R, *et al.*	2019	Alemanha	Revisão Sistemática	How effective and safe is medical cannabis as a treatment of mental disorders? A systematic review	Outra ferramenta	Medicamentos à base de THC e CBD foram associados à melhora de vários sintomas de transtornos mentais, mas não à remissão. A confiança geral nas evidências é baixa. São necessários mais estudos de alta qualidade metodológica e amostras grandes.	Outras	2
Nielsen S, Murnion B, Campbell G, Young H, Hall W.	2019	Inglaterra	Revisão Sistemática	Cannabinoids for the treatment of spasticity	GRADE	Atualmente, não há evidências suficientes para apoiar o uso de canabinoides no tratamento da espasticidade em	Outras	1

						crianças. São necessários mais estudos.		
Wang J, Wang Y, Tong M, *et al.*	2019	Estados Unidos	Revisão Sistemática e meta-análise	Medical Cannabinoids for Cancer Cachexia: A Systematic Review and Meta-Analysis	Rob Tools (Cochrane)	A análise mostrou que o canabinoide é eficaz no aumento do apetite em pacientes com câncer. No entanto, ele reduz a qualidade de vida, o que pode ser devido aos efeitos colaterais do canabinoide.	Neoplasias	3
Boland EG, Bennett MI, Allgar V, Boland JW.	2020	Inglaterra	Revisão Sistemática e meta-análise	Cannabinoids for adult cancer-related pain: systematic review and meta-analysis	Rob Tools (Cochrane)	Os canabinoides não podem ser recomendados para o tratamento da dor relacionada ao câncer, face a evidências com baixo risco de viés.	Dor	1
De Carvalho Reis R, Almeida KJ, da Silva Lopes L, *et al.*	2020	Estados Unidos	Revisão Sistemática e meta-análise	Efficacy and adverse event profile of cannabidiol and medicinal cannabis for treatment-resistant epilepsy: Systematic review and meta-analysis	Outra ferramenta	Este estudo indicou que o tratamento com CBD para epilepsia é eficaz na redução da frequência de convulsões.	Epilepsia	3
Elliott J, DeJean D, Clifford T, *et al.*	2020	Inglaterra	Revisão Sistemática	Cannabis-based products for pediatric epilepsy: An updated systematic review	GRADE	Para estudos controlados com CBD os desfechos positivos relacionados à epilepsia tiveram certeza de evidência de muito baixa a moderada. Para estudos observacionais, esses desfechos tiveram certeza de evidência baixa. A maioria das	Epilepsia	1

						evidências disponíveis refere-se apenas ao CBD de grau farmacêutico. Esta é uma área de pesquisa ativa, e atualizações futuras incluirão evidências adicionais assim que estiverem disponíveis.		
Gazendam A, Nucci N, Gouveia K, *et al.*	2020	Canadá	Revisão Sistemática e meta-análise	Cannabinoids in the Management of Acute Pain: A Systematic Review and Meta-analysis	GRADE	Há evidências de baixa qualidade indicando que os canabinoides podem ser uma alternativa segura para uma redução pequena, mas significativa, na pontuação subjetiva de dor no tratamento da dor aguda, com a administração intramuscular resultando em uma redução maior em relação à administração oral.	Dor	1
Hindocha C, Cousijn J, Rall M, Bloomfield MAP.	2020	Estados Unidos	Revisão Sistemática	The Effectiveness of Cannabinoids in the Treatment of Posttraumatic Stress Disorder (PTSD): A Systematic Review	CONSORT	Não se pode recomendar uso de canabinoides para sintomas globais de SEPT, distúrbios do sono e pesadelos na prática clínica, face aos resultados de estudos clínicos cuja qualidade foi avaliada.	Outras	1
Johal H, Devji T, Chang Y, *et al.*	2020	Canadá	Revisão Sistemática e meta-análise	Cannabinoids in Chronic Non-Cancer Pain: A Systematic Review and Meta-Analysis	GRADE	Houve evidência de qualidade moderada de pequeno efeito para o uso de canabinoides no tratamento de dor crônica não	Dor	2

						oncológica em todos os momentos estudados até 6 meses. Vale ressaltar que muitas condições podem ser classificadas como "dor crônica não oncológica" e a base de evidências sobre esse tópico é representada por certas condições mais do que por outras. São necessários mais estudos de qualidade.		
Khan R, Naveed S, Mian N, *et al.*	2020	Inglaterra	Revisão Sistemática	The therapeutic role of Cannabidiol in mental health: a systematic review	GRADE	Uso de CBD e nabiximols (que contêm CBD) é favorecido para psicose e esquizofrenia em Parkinson, transtorno de ansiedade social e comorbidades de TEA e TDAH, com recomendação moderada. Há evidências mais fracas para insônia, ansiedade, transtorno bipolar, SEPT e síndrome de Tourette.	Parkinson	2
							Ansiedade	2
							TEA	2
							TDAH	2
							Outros	1
Larsen C, Shahinas J.	2020	Canadá	Revisão Sistemática	Dosage, Efficacy and Safety of Cannabidiol Administration in Adults: A Systematic Review of Human Trials	GRADE	Há algumas evidências, embora de baixa qualidade, que apoiam o efeito ansiolítico da administração aguda de CBD oral. Há evidências de qualidade moderada	Outras	2

						de que a administração crônica e aguda de CBD pode melhorar os sintomas psicóticos em pacientes com esquizofrenia.		
Mun CJ, Letzen JE, Peters EN, *et al.*	2020	Estados Unidos	Revisão Sistemática	Cannabinoid effects on responses to quantitative sensory testing among individuals with and without clinical pain: a systematic review	Outra ferramenta	Em consonância com revisões anteriores, a revisão sistemática encontrou baixa consistência nos achados sobre a eficácia dos canabinoides como analgésicos.	Dor	1
Okusanya BO, Asaolu IO, Ehiri JE, *et al.*	2020	Inglaterra	Revisão Sistemática	Medical cannabis for the reduction of opioid dosage in the treatment of non câncer chronic pain: a systematic review	Rob Tools (Cochrane)	As evidências desta revisão não podem ser consideradas confiáveis para promover a *Cannabis* medicinal como um adjuvante aos opioides no tratamento da dor crônica não oncológica.	Dor	1
Rabgay K, Waranuch N, Chaiyakunapruk N, *et al.*	2020	Estados Unidos	Revisão Sistemática e meta-análise	The effects of cannabis, cannabinoids, and their administration routes on pain control efficacy and safety: A systematic review and network meta-analysis	Rob Tools (Cochrane)	Os resultados de meta-análise de rede demonstram que a *Cannabis* e os canabinoides podem aliviar diferentes tipos de dor quando administrados por diferentes vias: THC/CBD e THC por via oromucosal para dores neuropáticas e oncológicas, THC por via oral para dor oncológica, *cannabis* seca	Dor	2

						padronizada por THC (SCT) por via inalatória para dor neuropática e extrato de *cannabis* padronizado por THC (SCET) por via oral para dor nociceptiva. SCT, SCET e THC são os melhores em reduções de dor neuropática, nociceptiva e oncológica, respectivamente.		
Rodriguez-Almaraz JE, Chang S, Clarke J, *et al.*	2020	Estados Unidos	Revisão Sistemática e meta-análise	A systematic review and meta-analysis examining the effects of cannabis and its derivatives in adults with malignant CNS tumors	GRADE	As evidências apresentadas aqui sugerem que a *Cannabis* não aumenta o risco de morte em pacientes com tumores malignos do SNC. Em vez disso, embora o efeito geral da *cannabis* e dos canabinoides nas taxas de sobrevivência em um ano não tenha sido estatisticamente significativo, a direção de tal efeito sugere um aumento nas taxas de sobrevivência em um ano em populações com câncer do SNC.	Neoplasias	2
Wong SSC, Chan WS, Cheung CW.	2020	Estados Unidos	Revisão Sistemática	Analgesic Effects of Cannabinoids for Chronic Non-cancer Pain: a Systematic Review and Meta-Analysis with Meta-	GRADE	A revisão sistemática e meta-análise sugerem que os canabinoides administrados por via inalatória, oral	Dor	2

				Regression		ou oromucosal reduzem a dor crônica não oncológica, neuropática e não neuropática. O efeito analgésico foi pequeno e pode não ser clinicamente significativo.		
Ahmed S, Roth RM, Stanciu CN, Brunette MF.	2021	Suíça	Revisão Sistemática	The Impact of THC and CBD in Schizophrenia: A Systematic Review	GRADE	Não há evidências suficientes sobre a capacidade do THC ou do CBD de impactar os sintomas e a cognição em pacientes com esquizofrenia, de modo que nenhum dos canabinoides deve ser recomendado para o tratamento.	Outras	1
Ayala L, Winter S, Byrne R, *et al.*	2021	Estados Unidos	Revisão Sistemática	Assessments and Interventions for Spasticity in Infants with or at High Risk for Cerebral Palsy: A Systematic Review	Outra ferramenta	Nenhuma avaliação de espasticidade foi validada para crianças menores de dois anos com ou em alto risco de Paralisias cerebrais, enfraquecendo todas as conclusões sobre intervenções.	Paralisias cerebrais	1
Bosnjak Kuharic D, Markovic D, Brkovic T, *et al.*	2021	Cochrane Database	Meta-análise	Cannabinoids for the treatment of dementia	GRADE	Não está claro se os canabinoides apresentam algum benefício ou prejuízo no tratamento da demência, pois a certeza das evidências é baixa, sendo muito baixa para análogos de THC.	Demências	1

Sainsbury B, Bloxham J, Hassan M, *et al.*	2021	Estados Unidos	Revisão Sistemática e meta-análise	Efficacy of cannabis-based medications compared to placebo for the treatment of chronic neuropathic pain: a systematic review with meta-analysis	GRADE	Intervenções com THC e THC/CBD proporcionaram uma melhora significativa na intensidade da dor e maior probabilidade de redução de 30% na dor, mas as evidências foram de qualidade moderada a baixa.	Dor	2
Brigo F, Jones K, Eltze C, Matricardi S.	2021	Cochrane Database	Revisão	Anti-seizure medications for Lennox-Gastaut syndrome	GRADE	A evidência para outros medicamentos anticonvulsivantes (incluindo CBD) adicionais (em conjunto com outros fármacos) para cessação ou redução geral de convulsões foi baixa a muito baixa.	Epilepsia	1
Hammond S, Erridge S, Mangal N, *et al.*	2021	Estados Unidos	Revisão Sistemática e meta-análise	The Effect of Cannabis-Based Medicine in the Treatment of Cachexia: A Systematic Review and Meta-Analysis.	GRADE	A revisão não encontrou evidências de alta qualidade para recomendar o uso de produtos à base de *Cannabis* para o tratamento da caquexia.	Neoplasias	1
Dykukha I, Malessa R, Essner U, Überall MA	2021	Inglaterra	Meta-análise	Nabiximols in Chronic Neuropathic Pain: A Meta-Analysis of Randomized Placebo-Controlled Trials	GRADE	Nabiximols foram superiores ao placebo na redução da dor neuropática crônica, com um tamanho de efeito pequeno (evidência moderada).	Dor	2
Landrigan J, Bessenyei K, Leitner D, *et al.*	2021	Canadá	Revisão Sistemática	A systematic review of the effects of cannabis on cognition in people with multiple	Outra ferramenta	A literatura existente sobre a co-ocorrência do uso de *Cannabis* e esclerose múltipla	Esclerose múltipla	1

				sclerosis		(EM) carece de evidências de alta qualidade para recomendar ou não terapias com *Cannabis* e canabinoides para pessoas com EM com base nos efeitos cognitivos.		
Nabata KJ, Tse EK, Nightingale TE, *et al.*	2021	Emirados Árabes Unidos	Revisão Sistemática	The Therapeutic Potential and Usage Patterns of Cannabinoids in People with Spinal Cord Injuries: A Systematic Review	Outra ferramenta	Pessoas com Lesões Medulares (LM) usam canabinoides tanto recreativamente quanto por seus efeitos terapêuticos, principalmente para dor e espasticidade. As evidências existentes sugerem que os canabinoides podem ajudar a reduzir a dor e a espasticidade em pessoas com LME, pelo menos a curto prazo, mas a significância clínica e a magnitude de seus efeitos parecem obscuras.	Outras	2
Noori A, Miroshnychenko A, Shergill Y, *et al.*	2021	Inglaterra	Revisão Sistemática e meta-análise	Opioid-sparing effects of medical cannabis or cannabinoids for chronic pain: a systematic review and meta-analysis of randomised and observational studies	GRADE	Com base em evidências de certeza moderada a alta, a adição de *Cannabis* medicinal à terapia com opioides em pacientes com dor oncológica crônica teve pouco ou nenhum efeito no alívio da dor ou nos distúrbios do sono. Os efeitos	Dor	1

						poupadores de opioides da *cannabis* medicinal para dor crônica permanecem incertos devido a evidências de certeza muito baixa.		
Rehman Y, Saini A, Huang S, *et al.*	2021	Estados Unidos	Revisão Sistemática	Cannabis in the management of PTSD: a systematic review	Rob Tools (Cochrane)	Há uma escassez de evidências examinando os benefícios e malefícios associados ao uso de *Cannabis* em pacientes com SEPT. As evidências atuais sobre o uso de *Cannabis* para gerenciar SEPT são limitadas e baseadas em evidências de baixa qualidade.	Outras	1
Rosager EV, Møller C, Sjögren M.	2021	Alemanha	Revisão Sistemática	Treatment studies with cannabinoids in anorexia nervosa: a systematic review	Rob Tools (Cochrane)	Para ganho de peso em anorexia nervosa, o nível de evidência é baixo, visto que existem apenas dois ECRs com desenhos, tipos de canabinoides e níveis de exposição diferentes.	Outras	1
Simon L, Baldwin C, Kalea AZ, Slee A.	2021	Alemanha	Revisão Sistemática e meta-análise	Cannabinoid interventions for improving cachexia outcomes in cancer: a systematic review and meta-analysis	GRADE	Sem evidências de alta qualidade, nenhuma recomendação pode ser feita para apoiar o uso de canabinoides isoladamente para melhorar os sintomas e desfechos em pacientes com caquexia associada	Neoplasias	1

						ao câncer (CAC).		
Spanagel R, Bilbao A.	2021	Alemanha	Revisão Sistemática	Approved cannabinoids for medical purposes - Comparative systematic review and meta-analysis for sleep and appetite	GRADE	Os canabinoides não afetam negativamente o sono, embora o grau de evidência para essa conclusão seja muito baixo. Há evidência moderada de que os canabinoides podem diminuir o apetite (exclusivamente pelo CBD).	Outras	2
Steardo L Jr, Carbone EA, Menculini G, *et al.*	2021	Suíça	Revisão Sistemática	Endocannabinoid System as Therapeutic Target of PTSD: A Systematic Review	GRADE	Para SEPT, embora haja um racional robusto para o tratamento com medicamentos que atuam, direta ou indiretamente, no sistema canabinoide, e os resultados até o momento sejam promissores, mais estudos são necessários para investigar o perfil de segurança e eficácia de seu uso prolongado.	Outras	2
Thanabalasingam SJ, Ranjith B, Jackson R, Wijeratne DT.	2021	Inglaterra	Revisão Sistemática e meta-análise	Cannabis and its derivatives for the use of motor symptoms in Parkinson's disease: a systematic review and meta-analysis	Rob Tools (Cochrane)	A revisão encontrou evidências insuficientes para apoiar a integração da *Cannabis* medicinal na prática clínica da doença de Parkinson para o tratamento de sintomas motores, validando os resultados de revisões publicadas anteriormente. A maioria das	Parkinson	1

						evidências disponíveis foi avaliada como tendo alto risco de viés.		
Treves N, Mor N, Allegaert K, *et al.*	2021	Inglaterra	Revisão Sistemática e meta-análise	Efficacy and safety of medical cannabinoids in children: a systematic review and meta-analysis	Rob Tools (Cochrane)	O CBD demonstrou eficácia no tratamento da epilepsia na população pediátrica.	Epilepsia	3
Jugl S, Okpeku A, Costales B, *et al.*	2021	Suíça	Revisão de escopo	A Mapping Literature Review of Medical Cannabis Clinical Outcomes and Quality of Evidence in Approved Conditions in the USA from 2016 to 2019	AMSTAR ou AMSTAR-2	Em 11 condições, foram identificados poucos estudos com rigor e qualidade substanciais para contribuir com a base de evidências. No entanto, existem algumas condições para as quais evidências significativas sugerem que certas formas farmacêuticas e vias de administração de produtos de *Cannabis* medicinal provavelmente apresentam relações risco-benefício favoráveis. Lacunas nas evidências permanecem significativas para a maioria das condições, mas foram identificados vários registros de ECR sugerindo uma melhora no cenário das evidências nos próximos anos.	Outras	2
Vivace BJ, Sanders AN, Glassman SD, *et al.*	2021	Estados Unidos	Revisão Sistemática e meta-análise	Cannabinoids and orthopedic surgery: a systematic review	GRADE	Há dados escassos sobre o uso de canabinoides em	Dor	1

				of therapeutic studies		cirurgia ortopédica.		
Wang L, Hong PJ, May C, *et al.*	2021	Inglaterra	Revisão Sistemática e meta-análise	Medical cannabis or cannabinoids for chronic non-cancer and cancer related pain: a systematic review and meta-analysis of randomised clinical trials	GRADE	Evidências de moderada a alta certeza mostram que a *Cannabis* medicinal ou os canabinoides não inalados resultam em uma melhora pequena a muito pequena no alívio da dor, no funcionamento físico e na qualidade do sono em pacientes com dor crônica.	Dor	2
AminiLari M, Wang L, Neumark S, *et al.*	2022	Estados Unidos	Revisão Sistemática e meta-análise	Medical cannabis and cannabinoids for impaired sleep: a systematic review and meta-analysis of randomized clinical trials	GRADE	*Cannabis* medicinal provavelmente resulta em uma pequena melhora na qualidade do sono em comparação com placebo em pacientes com dor crônica, com evidências de certeza moderada. Para qualidade do sono, há evidência moderada a alta para ganhos pequenos em dor não oncológica e para ganhos muito pequenos em dor oncológica.	Outras	2
Okusanya BO, Lott BE, Ehiri J, *et al.*	2022	Suíça	Revisão	Medical Cannabis for the Treatment of Migraine in Adults: A Review of the Evidence	Outra ferramenta	Há algumas evidências do efeito benéfico da *Cannabis* medicinal no tratamento da enxaqueca em adultos. Mais estudos bem desenhados são	Dor	2

						necessários.		
Bahji A, Breward N, Duff W, *et al.*	2022	Estados Unidos	Revisão Sistemática	Cannabinoids in the management of behavioral, psychological, and motor symptoms of neurocognitive disorders: a mixed studies systematic review	Outra ferramenta	A revisão sistemática revelou uma escassez de estudos para uso de canabinoides nos transtornos neurocongnitivos.	Outras	2
Bialas P, Fitzcharles MA, Klose P, Häuser W.	2022	Inglaterra	Revisão Sistemática e meta-análise	Long-term observational studies with cannabis-based medicines for chronic non-cancer pain: A systematic review and meta-analysis of effectiveness and safety	GRADE	Os resultados desta revisão corroboram as recomendações mais específicas do documento de posicionamento da Federação Europeia da Dor, de que os produtos de *Cannabis* medicinal não podem ser usados em pacientes com dor crônica por falta de evidências robustas. No entanto, pacientes devidamente selecionados e supervisionados, dentro de uma abordagem de tratamento multicomponente, quando as opções de tratamento estabelecidas falharam, podem ter benefício.	Dor	2
Bilbao A, Spanagel R.	2022	Inglaterra	Revisão Sistemática e meta-análise	Medical cannabinoids: a pharmacology-based systematic review and meta-analysis for all relevant medical indications	GRADE	O CBD tem um efeito terapêutico significativo para epilepsia (grau alto) e parkinsonismo (grau moderado). Há evidências moderadas para o	Epilepsia	3
							Parkinson	2
							Dor	2
							Outros	1

						dronabinol na dor crônica, apetite e Tourette e evidências moderadas para nabiximols na dor crônica, espasticidade, sono e transtornos por abuso de substâncias. Todos os outros efeitos terapêuticos significativos têm grau de evidência baixo, muito baixo ou mesmo nenhum.		
C Votrubec, P Tran, A Lei, *et al.*	2022	Austrália	Revisão Sistemática	Cannabinoid therapeutics in orofacial pain management: a systematic review	Outra ferramenta	Há uma evidência de baixa qualidade que apoia o uso de canabinoides para tratar dor e inflamação, com uma falta de evidências de alta qualidade consistentes e convincentes relativas à sua eficácia na dor orofacial. Há evidências insuficientes para apoiar um benefício clínico tangível de canabinoides naturais e sintéticos no gerenciamento da dor orofacial, especialmente para medicamentos administrados na circulação sistêmica.	Dor	1
De Aquino JP, Bahji A, Gómez O, Sofuoglu M.	2022	Estados Unidos	Revisão Sistemática	Alleviation of opioid withdrawal by cannabis and	GRADE	Os resultados fornecem evidências preliminares de que,	Outras	1

				delta-9-tetrahydrocannabinol: A systematic review of observational and experimental human studies		embora a *Cannabis* e o THC possam aliviar a abstinência de opioides, esses efeitos provavelmente têm uma janela terapêutica estreita. Além disso, os efeitos dos canabinoides no alívio da abstinência podem depender do tipo de agonista opioide, dos níveis basais de exposição a opioides e canabinoides, de fatores individuais e do tipo de *Cannabis* e do canabinoide constituinte. Mais estudos são necessários para determinar o risco/benefício.		
Doppen M, Kung S, Maijers I, *et al.*	2022	Estados Unidos	Revisão Sistemática	Cannabis in Palliative Care: A Systematic Review of Current Evidence	GRADE	A qualidade das evidências avaliadas não respalda recomendações para o uso de *Cannabis* medicinal no contexto de cuidados paliativos, sendo a evidência muito baixa a baixa.	Outras	1
Filippini G, Minozzi S, Borrelli F, *et al.*	2022	Cochrane Database	Revisão Sistemática	Cannabis and cannabinoids for symptomatic treatment for people with multiple sclerosis	GRADE	Para EM, os nabiximols provavelmente reduzem a gravidade da espasticidade no curto prazo. A certeza da evidência está limitada pela curta duração dos estudos incluídos.	Esclerose múltipla	2

Fliegel DK, Lichenstein SD.	2022	Holanda	Revisão Sistemática	Systematic literature review of human studies assessing the efficacy of cannabidiol for social anxiety	Rob Tools (Cochrane)	Dados existentes sugerem que a administração aguda de CBD atenua significativamente a ansiedade social, sem sedação ou comprometimento cognitivo significativos. Mais estudos são necessários para avaliar dose e efeitos de mais longo prazo.	Ansiedade e Depressão	3
Gedin F, Blomé S, Pontén M, *et al.*	2022	Estados Unidos	Revisão Sistemática e meta-análise	Placebo Response and Media Attention in Randomized Clinical Trials Assessing Cannabis-Based Therapies for Pain: A Systematic Review and Meta-analysis	Rob Tools (Cochrane)	Os resultados desta revisão sistemática e meta-análise sugerem que as respostas ao placebo contribuem significativamente para a redução da dor em ensaios clínicos com canabinoides. A atenção excepcionalmente alta da mídia em torno dos ensaios com canabinoides, com relatos positivos independentemente dos resultados científicos, pode sustentar altas expectativas e moldar as respostas ao placebo em ensaios futuros.	Dor	1
Giossi R, Carrara F, Padroni M, *et al.*	2022	Nova Zelândia	Revisão Sistemática e meta-análise	Systematic Review and Meta-analysis Seem to Indicate that Cannabinoids for Chronic Primary	Rob Tools (Cochrane)	A qualidade da evidência foi em geral baixa a muito baixa, principalmente pela	Dor	1

				Pain Treatment Have Limited Benefit		imprecisão devido ao tamanho limitado da amostra e ao risco de viés. O tratamento geral com canabinoides em pacientes com dor primária crônica (DPC) teve benefício limitado no alívio da dor, com qualidade de evidência geralmente baixa.		
Quintero JM, Pulido G, Giraldo LF, *et al.*	2022	Colômbia	Revisão Sistemática	A Systematic Review on Cannabinoids for Neuropathic Pain Administered by Routes Other than Oral or Inhalation	Rob Tools (Cochrane)	A revisão sistemática da literatura revelou que há uma significativa falta de evidências sobre o papel de produtos canabinoides alternativos orais e inalatórios no tratamento da dor neuropática. Não é possível determinar a eficácia, tolerabilidade e segurança dos canabinoides administrados por essas vias.	Dor	1
McDonagh MS, Morasco BJ, Wagner J, *et al.*	2022	Estados Unidos	Revisão Sistemática	Cannabis-Based Products for Chronic Pain : A Systematic Review	Rob Tools (Cochrane)	Intervenções com *Cannabis* oral, sintética, com alta proporção de THC/CBD, e sublingual, extraída da planta e com proporção comparável de THC/CBD, podem estar associadas a melhorias a curto prazo na dor	Dor	2

						crônica, principalmente neuropática. As evidências sobre outros produtos foram insuficientes ou inexistentes.		
Nielsen S, Picco L, Murnion B, *et al.*	2022	Inglaterra	Revisão Sistemática e meta-análise	Opioid-sparing effect of cannabinoids for analgesia: an updated systematic review and meta-analysis of preclinical and clinical studies	GRADE	Estudos pré-clínicos e observacionais demonstram os potenciais efeitos poupadores de opioides dos canabinoides no contexto da analgesia, com evidência muita baixa, em contraste com ECRs de maior qualidade que não forneceram evidências de efeitos poupadores de opioides. Há alta evidência de baixo efeito.	Dor	1
Bialas P, Fitzcharles MA, Klose P, Häuser W.	2022	Inglaterra	Revisão Sistemática e meta-análise	Long-term observational studies with cannabis-based medicines for chronic non-cancer pain: A systematic review and meta-analysis of effectiveness and safety	GRADE	Para dor crônica não oncológica (DCNO) não está claro se há eficácia a longo prazo (dor, sono, humor, qualidade de vida relacionada à saúde), pois a certeza de evidência é muito baixa.	Dor	1
Pinto, J.S., Martel, F.	2022	Nova Zelândia	Revisão Sistemática	Effects of Cannabidiol on Appetite and Body Weight: A Systematic Review	Rob Tools (Cochrane)	À luz das evidências disponíveis, o CBD parece ter um efeito anorexígeno que pode ser mais substancial em indivíduos com índice de massa corporal (IMC) mais	Outras	2

						alto. Sabe-se que os canabinoides têm um efeito orexígeno mediado pelos receptores CB1 e CB2, mas, diferentemente do Δ9-THC, que possui efeito orexígeno, o CBD parece diminuir a ingestão alimentar.		
Price RL, Charlot KV, Frieler S, *et al.*	2022	Estados Unidos	Revisão Sistemática	The Efficacy of Cannabis in Reducing Back Pain: A Systematic Review	GRADE	No geral, há evidências crescentes de que a *Cannabis* pode ser eficaz no tratamento da dor nas costas; no entanto, dado o nível atual de evidências, não é possível concluir, neste momento, que a utilização rotineira da *Cannabis* seja uma alternativa aos opioides.	Dor	2
Silvinato A, Floriano I, Bernardo WM.	2022	Brasil	Revisão Sistemática e meta-análise	Use of cannabidiol in the treatment of epilepsy: Lennox-Gastaut syndrome, Dravet syndrome, and tuberous sclerosis complex.	GRADE	O uso de CBD em pacientes com epilepsias em comparação com placebo, mostra uma redução absoluta na frequência de convulsões e na eliminação de convulsões, com qualidade de evidência moderada. Mostra uma redução relativa na frequência de convulsões com alta qualidade de evidência.	Epilepsia	3
Urbi B, Corbett J, Hughes I, *et al.*	2022	Estados	Revisão	Effects of Cannabis	Rob Tools	Esta revisão não	Parkinson	1

		Unidos	Sistemática e meta-análise	in Parkinson's Disease: A Systematic Review and Meta-Analysis	(Cochrane)	encontrou evidências sólidas do uso benéfico da *Cannabis* em pacientes com doença de Parkinson. Nenhuma melhora clinicamente significativa nos sintomas gerais da doença de Parkinson foi detectada em estudos robustos, embora estudos não randomizados e pesquisas tenham sugerido benefícios potenciais.		
Velzeboer R, Malas A, Boerkoel P, *et al.*	2022	Estados Unidos	Revisão Sistemática	Cannabis dosing and administration for sleep: a systematic review	Rob Tools (Cochrane)	Os efeitos dos produtos de *Cannabis* no sono permanecem amplamente pouco estudados, com dados conflitantes entre os estudos, e ensaios clínicos randomizados de alta qualidade limitados. Neste momento é muito cedo para recomendar formulações e regimes de dosagem ideais.	Outras	1
Vinci A, Ingravalle F, Bardhi D, *et al.*	2022	Suíça	Revisão Sistemática e meta-análise	Cannabinoid Therapeutic Effects in Inflammatory Bowel Diseases: A Systematic Review and Meta-Analysis of Randomized Controlled Trials	GRADE	As evidências sobre a eficácia dos canabinoides no desfecho primário (remissão da doença ou redução significativa dos sintomas) na doença inflamatória	Outras	1

						intestinal são mistas. Observou-se nos ECR que pacientes com doença inflamatória intestinal, não se beneficiaram da terapêutica com canabinoides de forma significativa, quando comparados ao placebo.		
Vuilleumier C, Scherbaum N, Bonnet U, Roser P.	2022	Suíça	Revisão Sistemática	Cannabinoids in the Treatment of Cannabis Use Disorder: Systematic Review of Randomized Controlled Trials	Rob Tools (Cochrane)	As evidências são, neste momento, muito fracas para apoiar qualquer medicamento específico em transtorno do uso de *Cannabis*.	Outras	1
Barakji J, Korang SK, Feinberg J, *et al.*	2023	Estados Unidos	Meta-análise	Cannabinoids versus placebo for pain: A systematic review with meta-analysis and Trial Sequential Analysis.	GRADE	Os canabinoides reduziram a dor crônica e melhoraram a qualidade do sono, não tiveram efeitos na dor aguda ou na dor oncológica, sempre com certeza baixa de evidência.	Dor	2
Belgers V, Röttgering JG, Douw L, *et al.*	2023	Estados Unidos	Meta-análise	Cannabinoids to Improve Health-Related Quality of Life in Patients with Neurological or Oncological Disease: A Meta-Analysis	Rob Tools (Cochrane)	A meta-análise de ECRs não demonstrou efeito dos canabinoides na qualidade de vida relacionada à saúde (QVRS) ou no bem-estar mental em pacientes com câncer ou doença do SNC.	Ansiedade e Depressão	1
Chou R, Wagner J, Ahmed AY, *et al.*	2023	Estados Unidos	Revisão Sistemática	Living Systematic Review on Cannabis and Other Plant-Based Treatments for Chronic Pain:	GRADE	Há apenas evidências de curto prazo disponíveis para intervenções relacionadas à	Dor	2

				Surveillance Report 4: Literature Update Period: Mid-January 2022 Through March 2022		*Cannabis* contendo THC e/ou CBD para tratar principalmente dor crônica neuropática. A melhora na dor foi pequena a moderada com produtos com alta e comparável proporção de THC para CBD.		
Häuser W, Welsch P, Radbruch L, *et al.*	2023	Cochrane Database	Revisão	Cannabis-based medicines and medical cannabis for adults with cancer pain	GRADE	Nabiximols e THC são ineficazes no alívio da dor oncológica moderada a grave refratária a opioides, com evidência moderada. Não está claro se a nabilona é eficaz na redução da dor associada a tratamentos de câncer de cabeça e pescoço e câncer de pulmão de células não pequenas; se uma dose única de análogos sintéticos de THC é superior a um equivalente de morfina em dose baixa na redução da dor oncológica de moderada a grave; se CBD agrega valor aos cuidados paliativos especializados isoladamente na redução da dor em pessoas com câncer avançado, pois a certeza da evidência é baixa.	Dor	1
McParland AL, Bhatia A, Matelski J, *et*	2023	Inglaterra	Revisão	Evaluating the	GRADE	Os canabinoides	Dor	3

al.			Sistemática e meta-análise	impact of cannabinoids on sleep health and pain in patients with chronic neuropathic pain: a systematic review and meta-analysis of randomized controlled trials		desempenham um papel importante no tratamento da dor neuropática crônica, como evidenciado por melhorias significativas na qualidade do sono, e na intensidade e na percepção da dor. Mais estudos são necessários.		
Murni W, Umar TP, Tandarto K, *et al.*	2023	Brasil	Revisão Sistemática	Efficacy and safety of medical cannabinoids in children with cerebral palsy: a systematic review	Rob Tools (Cochrane)	Os canabinoides podem ser benéficos em pacientes com paralisia cerebral; no entanto, sua eficácia ainda precisa ser exaustivamente estudada.	Paralisias cerebrais	2
Ranum RM, Whipple MO, Croghan I, *et al.*	2023	Estados Unidos	Revisão Sistemática	Use of Cannabidiol in the Management of Insomnia: A Systematic Review	Outra ferramenta	CBD, isoladamente ou em quantidades equivalentes de THC, pode ser benéfico no alívio dos sintomas de insônia.	Outras	2
Solmi M, De Toffol M, Kim JY, *et al.*	2023	Inglaterra	Revisão Umbrella de meta-análises	Balancing risks and benefits of cannabis use: umbrella review of meta-analyses of randomised controlled trials and observational studies.	GRADE	O CBD mostrou efetividade em eventos convulsivos com certeza alta da evidência. Medicamentos à base de *Cannabis* ou canabinoides reduziram a dor, melhoraram o limiar de dor e desconforto, com certeza alta da evidência. Para esclerose múltipla, os canabinoides melhoraram a espasticidade e dor,	Epilepsia	3
							Dor	3
							Esclerose múltipla	3
							Neoplasia	2
							Outros	2

						com certeza moderada da evidência. Para câncer, os canabinoides melhoraram a qualidade do sono, com certeza moderada.		
Strand NH, Maloney J, Kraus M, *et al.*	2023	Suíça	Revisão Sistemática	Cannabis for the Treatment of Fibromyalgia: A Systematic Review	GRADE	Para redução da dor a curto prazo em pessoas com fibromialgia, tratadas com terapias canabinoides, as evidências que sustentam o uso são de baixa qualidade.	Dor	1
Longo R, Oudshoorn A, Befus D.	2021	Estados Unidos	Revisão Sistemática	Cannabis for Chronic Pain: A Rapid Systematic Review of Randomized Control Trials	Oxford Quality score ou Jadad scale	As evidências atuais representam um desafio para concluir a eficácia da *Cannabis* para dor crônica devido a resultados inconsistentes entre os ECRs.	Dor	1

Conclusões para prática clínica:

(1) evidência de qualidade muito baixa ou baixa/de nível baixo, por alto risco de viés dos estudos/sem recomendação de emprego clínico, ou texto indicativo de não adoção na prática clínica;

(2) evidência de qualidade moderada-baixa baseada em poucos estudos de baixa qualidade metodológica, ou inconclusiva, ou mesmo relato de falta de evidências, ou de insuficiente número de estudos;

(3) evidências de qualidade alta ou moderada-alta, ou com nível alto, por baixo risco de viés/com recomendação de emprego clínico ou texto claramente favorável à intervenção.
